# Supplementary material for: The Systems Biology Research Tool: evolvable open-source software
Source: BMC Syst Biol. 2008 Jun 29;2:55. doi: 10.1186/1752-0509-2-55 (PMC2446383; doi:10.1186/1752-0509-2-55)
Supplement: Additional file 1 — SBRT Archive. An archive of the current version of the Systems Biology Research Tool. [file 1752-0509-2-55-S1.zip › sbrt-1.4.0/doc/developers_guide/api/sbrt/kernel/util/UnmodifiableMap.html]

UnmodifiableMap


|  |  |  |
| --- | --- | --- |
| **Overview** \| **Package** \| **Class** \| **Use** \| **Tree** \| **Deprecated** \| **Index** \| **Help** | |  |
| **PREV CLASS**   **NEXT CLASS** | **FRAMES**    **NO FRAMES**     **All Classes** |  |
| SUMMARY: NESTED \| FIELD \| CONSTR \| METHOD | DETAIL: FIELD \| CONSTR \| METHOD |  |


---


## sbrt.kernel.util Class UnmodifiableMap<K,V>

```
java.lang.Object
  sbrt.kernel.util.UnmodifiableMap<K,V>
```

**Type Parameters:**: `K` - the key type.: `V` - the value type.

**All Implemented Interfaces:**: java.util.Map<K,V>

---

``` public class UnmodifiableMap<K,V> extends java.lang.Object implements java.util.Map<K,V> ```

This class is used to create an unmodifiable view of maps. It
is intended to mimic the behavior of
`java.util.Collections.unmodifiableMap(Map)`,
but in a more explicit way. If a method returns an
`UnmodifiableMap`, it's clear that the map is
unmodifiable and that `UnsupportedOperationException` will be thrown
if modification is attempted.

**Author:**
:   This class was written and documented by Jeremiah Wright while
    in the Wagner lab.

---

**Nested Class Summary**

**Nested classes/interfaces inherited from interface java.util.Map**

`java.util.Map.Entry<K,V>`


**Constructor Summary**

`UnmodifiableMap(java.util.Map<? extends K,? extends V> m)`
  
          Constructs a new unmodifiable view of the provided map.


| **Method Summary** | |
| --- | --- |
| `void` | `clear()`             See Map.clear(). |
| `boolean` | `containsKey(java.lang.Object key)`             See Map.containsKey(Object). |
| `boolean` | `containsValue(java.lang.Object value)`             See Map.containsValue(Object). |
| `UnmodifiableSet<java.util.Map.Entry<K,V>>` | `entrySet()`             See Map.entrySet(). |
| `boolean` | `equals(java.lang.Object obj)`             See Map.equals(Object). |
| `V` | `get(java.lang.Object key)`             See Map.get(Object). |
| `int` | `hashCode()`             See Map.hashCode(). |
| `boolean` | `isEmpty()`             See Map.isEmpty(). |
| `UnmodifiableSet<K>` | `keySet()`             See Map.keySet(). |
| `V` | `put(K key, V value)`             See Map.put(Object, Object). |
| `void` | `putAll(java.util.Map<? extends K,? extends V> t)`             See Map.putAll(Map). |
| `V` | `remove(java.lang.Object key)`             See Map.remove(Object). |
| `int` | `size()`             See Map.size(). |
| `java.lang.String` | `toString()`             Returns `toString()` on the underlying map. |
| `UnmodifiableCollection<V>` | `values()`             See Map.values(). |

**Methods inherited from class java.lang.Object**

`clone, finalize, getClass, notify, notifyAll, wait, wait, wait`

**Constructor Detail**

### UnmodifiableMap

```
public UnmodifiableMap(java.util.Map<? extends K,? extends V> m)
```

:   Constructs a new unmodifiable view of the provided map.

    **Parameters:**: `m` - the map for which an unmodifiable view is created.


**Method Detail**

### clear

```
public void clear()
```

:   See Map.clear().

    :   **Specified by:**: `clear` in interface `java.util.Map<K,V>`

---


### containsKey

```
public boolean containsKey(java.lang.Object key)
```

:   See Map.containsKey(Object).

    :   **Specified by:**: `containsKey` in interface `java.util.Map<K,V>`

---


### containsValue

```
public boolean containsValue(java.lang.Object value)
```

:   See Map.containsValue(Object).

    :   **Specified by:**: `containsValue` in interface `java.util.Map<K,V>`

---


### entrySet

```
public UnmodifiableSet<java.util.Map.Entry<K,V>> entrySet()
```

:   See Map.entrySet().

    :   **Specified by:**: `entrySet` in interface `java.util.Map<K,V>`

---


### get

```
public V get(java.lang.Object key)
```

:   See Map.get(Object).

    :   **Specified by:**: `get` in interface `java.util.Map<K,V>`

---


### isEmpty

```
public boolean isEmpty()
```

:   See Map.isEmpty().

    :   **Specified by:**: `isEmpty` in interface `java.util.Map<K,V>`

---


### keySet

```
public UnmodifiableSet<K> keySet()
```

:   See Map.keySet().

    :   **Specified by:**: `keySet` in interface `java.util.Map<K,V>`

---


### put

```
public V put(K key,
             V value)
```

:   See Map.put(Object, Object).

    :   **Specified by:**: `put` in interface `java.util.Map<K,V>`

---


### putAll

```
public void putAll(java.util.Map<? extends K,? extends V> t)
```

:   See Map.putAll(Map).

    :   **Specified by:**: `putAll` in interface `java.util.Map<K,V>`

---


### remove

```
public V remove(java.lang.Object key)
```

:   See Map.remove(Object).

    :   **Specified by:**: `remove` in interface `java.util.Map<K,V>`

---


### size

```
public int size()
```

:   See Map.size().

    :   **Specified by:**: `size` in interface `java.util.Map<K,V>`

---


### values

```
public UnmodifiableCollection<V> values()
```

:   See Map.values().

    :   **Specified by:**: `values` in interface `java.util.Map<K,V>`

---


### toString

```
public java.lang.String toString()
```

:   Returns `toString()` on the underlying map.

    :   **Overrides:**: `toString` in class `java.lang.Object`
    :   **Returns:**: `toString()` on the underlying map.

---


### equals

```
public boolean equals(java.lang.Object obj)
```

:   See Map.equals(Object).

    :   **Specified by:**: `equals` in interface `java.util.Map<K,V>` **Overrides:**: `equals` in class `java.lang.Object`

---


### hashCode

```
public int hashCode()
```

:   See Map.hashCode().

    :   **Specified by:**: `hashCode` in interface `java.util.Map<K,V>` **Overrides:**: `hashCode` in class `java.lang.Object`


---


|  |  |  |
| --- | --- | --- |
| **Overview** \| **Package** \| **Class** \| **Use** \| **Tree** \| **Deprecated** \| **Index** \| **Help** | |  |
| **PREV CLASS**   **NEXT CLASS** | **FRAMES**    **NO FRAMES**     **All Classes** |  |
| SUMMARY: NESTED \| FIELD \| CONSTR \| METHOD | DETAIL: FIELD \| CONSTR \| METHOD |  |


---
